# Role of breathing training programs on quality of life in chronic kidney disease patients

**DOI:** 10.3934/publichealth.2023029

**Published:** 2023-05-11

**Authors:** Ana I. Rubio-López, Alejandro Heredia-Ciuró, Jorge L. Marin-DelaRosa, Javier Martín-Núñez, María Granados-Santiago, María C. De Gracia-Guindo, Marie C. Valenza

**Affiliations:** 1 Department of Physiotherapy, Faculty of Health Sciences, University of Granada, Granada, Spain; 2 Department of Nephrology, Virgen de las Nieves University Hospital, Granada, Spain

**Keywords:** breathing training, chronic kidney disease, kidney failure, hemodialysis, quality of life

## Abstract

**Background:**

Due to its chronic and progressive nature, chronic kidney disease (CKD) affects patients in many spheres including their perception of quality of life (QOL). Breathing training techniques have shown positive effects on health and QOL for different conditions.

**Objective:**

The aim of this study was to perform a scoping review to examine the characteristics related to the application of breathing training on patients with CKD, and to identify the relevant outcomes and target group for the application of breathing training.

**Methods:**

This scoping review was performed in accordance with PRISMA-SRc guidelines. We systematically searched three electronic databases for articles published before March 2022. The studies included patients with chronic kidney disease that received breathing training programs. The breathing training programs were compared to usual care or no treatment.

**Results:**

A total of four studies were included in this scoping review. The four studies had heterogeneous disease stages and breathing training programs. All the studies included reported positive effects of breathing training programs on QOL of CKD patients.

**Conclusion:**

The breathing training programs were able to improve the quality of life of patients with CKD undergoing hemodialysis treatment.

## 1. Introduction

Chronic kidney disease (CKD) is a healthcare burden due to the high economic costs it generates for health systems and its high incidence and prevalence. The mortality of CKD has increased in the last 10 years. It is currently the 12th most common cause of death according to the Global Burden of Disease Study in 2015 and also one of the fastest rising major causes of mortality, along with diabetes and dementia [1]–[3]. CKD is defined as abnormalities of kidney structure or function present for over 3 months with specific implications for health [4]. An expanded definition of CKD includes a glomerular filtration rate of less than 60 mL/min/1.73m^2^ and a 1-time urine albumin-creatinine ratio of at least 30 mg/g with or without kidney damage, or more markers of kidney failure [4]–[6]. The clinical progression of the disease is described in 5 stages. In the most advanced stage, kidney replacement therapy is proposed to patients in the form of hemodialysis or peritoneal dialysis [7].

CKD is associated with many health consequences, including metabolic, endocrine and cardiovascular alterations. It is also strongly associated with pulmonary edema and respiratory muscle dysfunction, leading to a high risk of lung dysfunction in affected patients. The prevalence of lung dysfunction increases in CKD patients from stages 1 to 4 according to the National Health and Nutrition Examination Survey (NHANES) 2007–2012 [8],[9].

Due to its progressive nature, CKD affects patients in many spheres along the course of the disease including physical, mental and emotional well-being. It changes their daily living and social participation and decreases their perception of quality of life (QOL). Patients with CKD have to change their lifestyles, habits and nutrition and adjust to medical treatments and physical limitations. They experience existential and emotional conflicts, among other health situations and biopsychosocial changes that negatively impact their QOL [7]. In addition, QOL is a marker of disease burden and the assessment of QOL is an important criterion of the effectiveness of many treatments and interventions in health care and a predictor for adverse outcomes [10],[11].

According to the World Health Organization (WHO) [12] and the American College of Sports Medicine [13], regular exercise training has been proven to help populations maintain healthy levels of quality of life, mitigating health risks. It is also considered safe for adults living with the selected chronic conditions.

Different exercise modes are very popular in the health and fitness industry at European [14] and global level [15]. Respiratory training has been included in trendy exercise modes and is currently used in a wide range of populations [16],[17]. In fact, breathing training techniques have shown positive effects on health in patients with different conditions such as chronic obstructive pulmonary disease (COPD), asthma, postoperative pulmonary function, and cardiorespiratory function, among others [18].

However, it is unclear what kind of information is available in the literature about the effects of breathing training on patients with CKD in need of dialysis treatment. For these reasons, a scoping review was conducted to systematically map the research conducted in this area and identify any existing gaps in knowledge.

The objectives of this study were to examine the characteristics related to the application of breathing training on patients with CKD, and to identify the relevant outcomes and target group for the application of breathing training. Additionally, this scoping review was aimed at developing and confirming our prior inclusion criteria to ensure that the question asked by the subsequent systematic review could be answered by available and relevant evidence.

## 2. Materials and methods

### 2.1. Study registration

This scoping review is reported following the Preferred Reporting Items for Scoping Reviews (PRISMA ScR) guidelines [19] and was registered in the Prospective Register of Systematic Reviews (PROSPERO) with identification number CRD42021288231. Additionally, we followed the method suggested by Arksey and O'Malley [20] as standard steps for the development of scoping reviews.

### 2.2. Research question

We applied the recommended use of the PCC mnemonic (Population, Concept and Context) to guide question development [21]. The inclusion criteria were (1) patients with chronic kidney disease (2) who received breathing training programs and (3) the breathing training intervention had to be compared to a control group that received usual care or no treatment.

### 2.3. Identifying relevant studies

We conducted a broad search of the literature for indexed articles on electronic databases MEDLINE/PubMed, Web of Science and Scopus from their inception to March 2022. The search strategy was designed using the following steps: (1) examining relevant key terms used in existing systematic reviews to develop our keywords, (2) a thorough search for terms in the MeSH Database, (3) and expert guidance by a specialist. The strategy was adapted to index across other databases. We screened the references of relevant reviews to screen for additional studies that could potentially be included in this scoping review. The full search strategy is shown in Appendix A.

### 2.4. Study selection

All the searched citations were stored in the Mendeley Desktop 1.19.4 reference manager application. Duplicated studies retrieved from electronic searches were removed. Two independent researchers screened the titles and abstracts of articles found in the searches (A.I.R., A.H.C.). Studies appearing to meet the inclusion criteria and those with insufficient data to make a clear decision were selected for evaluation of the full manuscript to determine their eligibility. Disagreement was solved by a third researcher (C.V.).

### 2.5. Charting the data

We charted key items of information obtained from the primary research reports reviewed. Data extraction was performed by one of the researchers through a custom-designed data extraction form created in Excel (Microsoft Corporation, Redmond, WA), using ‘data charting form’. This form included information on the study population, the type of intervention and the outcome measures employed. We recorded information as follows:

- Author(s), year of publication
- Design
- Pathology treatment status
- Study populations
- Intervention type, and comparator (if any); duration of the intervention
- Outcome measures
- Important results

### 2.6. Methodological quality

Two authors independently assessed the methodological quality and the risk of bias of individual studies. We used the Downs and Black Checklist [22] to assess methodological quality. This assessment method includes 27 items in five subscales (study quality, external validity, study bias, confounding and selection bias, and study power). It classifies the quality of studies as follows: excellent when scoring 26 or more points, good between 20 and 25 points, fair between 15 and 19, and poor when the score is 14 or less. Due to its high validity and reliability, this scale is one of the most suitable scales for use in research reviews [23],[24].

The risk of bias was assessed with the Cochrane risk-of-bias tool for randomized control trials [25]. The items of this tool classify the risk of bias as high when the methodological procedure is not described, unclear if the description is unclear, and low when the procedure is described in detail. A study is considered to have good quality when all criteria are met and fair quality when one criterion is not met or two criteria are unclear, and there is no known important limitation that could invalidate the results. It is considered to have poor quality when two or more criteria are listed as having high or unclear risk of bias, or when one criterion is not met or two criteria are unclear and there are important limitations that could invalidate the results [26].

## 3. Results:

### 3.1. Search results

**Figure 1.**
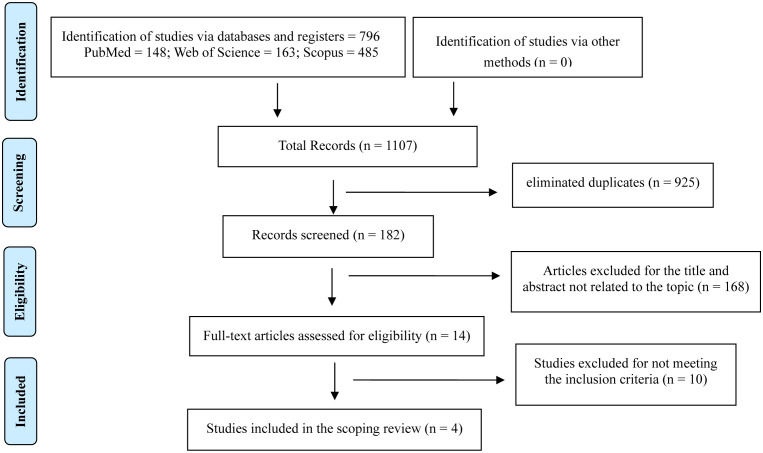
Flowchart.

Our search strategy identified 796 potentially eligible articles from MEDLINE/PubMed, Web of Science and Scopus databases. After removal of duplicates and studies with animals, 182 titles and abstracts were screened for potentially relevant articles. Fourteen studies were selected for full-text evaluation. Finally, 4 papers were included in the scoping review [27]–[30]. Details of the study selection procedure are listed in Figure 1.

### 3.2. Quality assessment

The results of the methodological quality of studies included are shown in Table 1 The risk of bias in all these studies ranged from 23 to 26 points. Only the study by Tsai et al. [27] had a good quality score, and the studies by Huang et al. [29] and Kharbteng et al. [28] had a poor quality score.

### 3.3. Study characteristics

A total of 206 subjects with CKD were assessed in the studies included, and 60.5% were male. The experimental groups included 92 patients aged between 52 and 66 years, and the control group included 86 patients aged between 51 and 61 years. Of the studies included, two were conducted in Taiwan [27],[29] and one was conducted in India [28]. The kidney disease stage of the patients was heterogeneous. One study included patients with CKD who received hemodialysis in two or three three-hour sessions weekly for more than three months [27], two studies [28],[30] included patients without kidney replacement therapy (KRT) who had a clinically stable course for the last month and an estimated glomerular filtration rate (GFR) between 14 and 45 ml/min/1.73m^2^ [27] and one study included patients with kidney failure undergoing hemodialysis treatment three times per week for at least three months [29].

### 3.4. Intervention

Details about applied interventions and obtained results are reported in Table 2. Breathing training programs were applied heterogeneously, that is, isolated or combined; three studies [27],[28],[30] applied isolated breathing training, and one study [29] combined breathing training and leg exercises.

The components of the usual care in the control groups were also heterogeneous. Tsai et al. [27] assigned patients in the control group to a waiting list and after the post-test measurements were completed, the control group received breathing training for four weeks. Kharbteng et al. [28] did not specify the usual care components. Huang et al. [29] described usual care including routine medications, medical treatment, and guidance regarding diet, daily activity and water restrictions.

The duration and timing of training also varied among studies. Tsai et al. [27] and Kaneko et al. [30] designed a four-week intervention program with breathing exercises, twice weekly for a total of eight sessions with no specifications of the timing related to treatment. The intervention used by Kharbteng et al. [28] consisted of 5-minute sessions three times a day for 4 weeks. Participants in the study by Huang et al. [29] underwent a 12-week intervention three times per week performed two hours after hemodialysis was initiated.

Outcome measures

Quality of life was the main outcome measure, and it was measured with different instruments in the studies analyzed. One study [27] assessed health-related quality of life using the Medical Outcome Studies 36-Short Form Health Survey (SF-36). Another study [28] measured quality of life with the Kidney Disease and Quality of Life questionnaire (KDQOL™-36). Finally, a study [29] used the Chinese version of the World Health Organization quality of life assessment brief to reflect quality of life and general health status.

There were also other outcomes measured in the studies included in this scoping review such as depression measured with the Beck Depression Inventory-II (BDI-II) and self-reported sleep quality assessed using the Pittsburg Sleep Quality Index (PSQI) [27], as well as heart rate variability and fatigue assessed with the hemodialysis-related fatigue scale [29],[30]. The study by Kaneko et al. [30] also assessed blood pressure, respiratory rate, skin temperature, and skin blood flow.

### 3.5. Results of individual studies

The analyzed studies showed significant improvements in quality of life after treatment intervention. Tsai et al. [27] reported that the intervention group had scores significantly higher than the control group for both the role-emotional subscale and the mental component summary of the 36-Item Short-Form Health Survey (SF-36). In their study, Kharbteng et al. [28] found a significant difference in mean scores in the intervention group for the KDQOL™-36 for the subscales effects of kidney disease, SF-12 physical functioning or physical health component, and SF-12 mental functioning or mental health composite. Huang et al. [29] found significant changes in quality of life after the intervention in the experimental group.

Additionally, the study by Tsai et al. [27] showed a significant decrease in depressive symptoms after treatment but no changes in sleep quality. Huang et al. [29] found significant decreases in fatigue but no significant changes in heart rate variability. The study by Kaneko et al. [30] also showed significant differences in diastolic BP, respiratory rate, skin temperature, HF, and the LF/HF ratio, after applying the breathing intervention. The details of interventions and obtained results are reported in Table 2.

**Table 1.** Characteristics of studies.

**Study (year)**	**Design**	**Pathology treatment status**	**Sample (% male)**	**Sample Age Years ± SD**	**Quality of assessment Downs and Black (risk of bias)**
Huang et al. (2021) [29]	RCT	KF In hemodialysis 3 times / week At least 3 months	**EG:** n = 40 (72.5%) **CG:** n = 43 (65.1%) **Total:** n = 83 (68.67%)	**EG:** 53.70 ± 10.04 **CG:** 61.19 ± 10.19	24 (Poor quality)
Kharbteng et al. (2020) [28]	RCT	CKD without KRT Clinically stable course for at least 1 month	**EG:** n = 30 (50%) **CG:** n = 30 (70%) **Total:** n = 60 (60%)	**EG:** 52.06 ± 6.97 **CG:** 51.83 ± 10.27	23 (Poor quality)
Tsai et al. (2015) [27]	RCT	CKD In hemodialysis 2/3 times / week 3 hours / season At least 3 months	**EG:** n = 32 (50%) **CG:** n = 25 (48%) **Total:** n = 57 (49.12%)	**EG:** 64.94 ± 9.51 **CG:** 61.08 ± 11.18	26 (Good quality)
Kaneko et al (2021) [30]	Pilot quasi-experimental study	CKD without KRT in a stable condition	**EG:** n = 6 (100%) **CG:** -	**EG:** 66.0 ± 9.4 **CG:** -	-

*Note: RCT – Randomized controlled trial; KF – Kidney failure; CKD – Chronic kidney disease; KRT – Kidney replacement therapy; EG – Experimental group; CG – Control group; SD – Standard deviation.

**Table 2.** Characteristics of interventions.

**Study (year)**	**Timing of intervention**	**Interventions**	**Outcomes**	**Main results**
**Huang et al. (2021) [29]**	During hemodialysis sessions (3 hours) 12 weeks 3 times/week	**EG** Usual care Breathing-based low-intensity leg exercise program leg lifts + quadriceps femoris contraction + knee flexion + five abdominal breaths 15 min/exercise section **CG** Usual care: routine medication, medical treatment and guidance (diet + daily activity + water restrictions)	- QOL WHOQOL_BREF - Heart rate variability Low-frequency power is associated with the clinical response to sympathetic and parasympathetic activity and high-frequency power, which is an index of parasympathetic activity. - Fatigue The hemodialysis-related fatigue scale.	↑ WHOQOL * ↓ Fatigue* LF X HF X
**Kharbteng et al. (2020) [28]**	At home 4 weeks 7 times / week 3 times / day	**EG** Breathing training program (alternate nostril breathing or anulom-vilom) 4-7-8 breathing exercises and breath counting 5 min/session (15 min/day) **CG** Usual care	- QOL KDQOL-36	↑KDQOL™-36*
**Tsai et al. (2015) [27]**	NR (at the dialysis center) 4 weeks 2 times / week	**EG** Audio device-guided breathing training 1st session: 10 min individualized breathing coaching Listening to prerecorded instructions on breathing technique 20 min practiced breathing + prerecorded voice guide 7 following sessions: 30 min listening to prerecorded voice guide and music + practicing breathing **CG** Waiting list After the posttest measurements were completed, patients received four weeks of breathing training	- QOL SF-36 - Depression BDI-II - Sleep quality PSQI	↓ BDI-II * PSQI X ↑Role-emotional subscale and mental component summary of QoL FS-36*
**Kaneko et al (2021) [30]**	NR Around 4 weeks 2 times / day	**EG** Six abdominal breaths per minute for 15 minutes Subjects repeatedly inhaled for 3 seconds through the nose and exhaled for 6 seconds through the mouth. **CG** No control group	-Heart rate -Blood pressure -Respiratory rate -Skin temperature -Skin blood flow -Heart rate variability: LF, HF, ratio of LF and HF power	HR X Systolic BP X ↓ Diastolic BP* ↓ Respiratory rate* ↑Skin temperature* Skin blood flow X LF X ↑ HF* ↓ LF/HF ratio*

*Note: EG – Experimental group; CG – Control group; NR – Not reported; QOL – Quality of life; WHOQOL_BREF – World Health Organization quality of life-brief version; LF – Low-frequency power; HF – High-frequency power; KDQOL-36 – Kidney Disease and Quality of Life questionnaire; SF-36 – Medical Outcome Studies 36-Item Short Form Health Survey; BDI-II – Beck Depression Inventory II; PSQI – Pittsburgh Sleep Quality Index; BP – Blood pressure; *: Statistically significant; ↑: Increment; ↓: Decrement; X: No statistically relevant variations.

## 4. Discussion

To our knowledge, this is the first scoping review to evaluate the effects of breathing training on patients with CKD treated by dialysis. The small number of included studies and the publication years indicate the novelty and limited research to date. Even with the heterogeneity of the studies included, our findings suggest that breathing training alone or combined with leg exercises has positive effects on quality of life in CKD patients without KRT or hemodialysis treatment.

Even the analysed studies used different approaches to breathing training program design and choice of technique; all of them included abdominal breathing, a breathing exercise that seemed to have positive effects on quality of life. The four included studies [27]–[30] followed similar coaching method for teaching their breathing training programs to participants in the experimental group. The experimental group in all the studies [27]–[30] received a coaching training demonstration by the researchers. In addition, to enhance the intervention performance, one study used pre-recorded instructions to guide each session [27]; in another two studies, the experimental group was guided with a video provided to each participant in the experimental group with the purpose of either standardizing the program and correcting the practice [29] or practicing the exercises at home [28]. This methodology could also have ensured adherence to treatment.

Even though the duration of the interventions and of the entire protocols were heterogeneous among the studies, the evidence in this scoping review suggests that a breathing training intervention as short as a total of 8 sessions in 4 weeks has positive benefits in some areas of the quality of life in CKD patients treated by hemodialysis [27],[30]. In this regard, other exercise types have demonstrated similar improvement in CKD quality of life and functional status, with greater values of TAC,CAT,GSH and GSH/GSSG after the exercise program [31].

Given that no special equipment was required, after coaching, respiratory training could be performed from home without taking much time, with good benefits as reported by Kharbteng et al. [28] This matches the findings of Lu et al. [32], which concluded that home-based breathing exercises have beneficial effects on chronic obstructive pulmonary disease.

The study by Tsai et al. [27] had self-reported depressive symptoms as primary outcome and the health-related quality of life and self-reported sleep quality as secondary outcomes. The latter outcome showed no statistically relevant variations, but the breathing program had positive and statistically relevant changes in the other two outcomes. Similarly, the study by Levendoglu et al. [33] showed a significant reduction of depression levels and the mental component scale of CKD patients after applying a twelve-week exercise program.

Other studies support our findings with breathing training as a promising intervention to improve health outcomes and quality of life in various pathologies such as heart failure [34] and chronic obstructive pulmonary disease [35].

### 4.1. Strengths

The strength of our study is that it is the first to review the effects of breathing training on CKD patients. Additionally, it includes research published about the topic to date.

### 4.2. Limitations

This scoping review has several limitations. Our analysis included a small number of studies; nevertheless, previous reviews have been conducted with a similar number of studies [36]. Additionally, the interventions of the studies included were not homogeneous, making it difficult to categorize the results.

## 5. Conclusions

In conclusion, a breathing training intervention for at least 4 weeks, including diaphragmatic breathing exercises, was able to improve the quality of life of patients with CKD during hemodialysis treatment.

These findings could improve the daily clinical practice of CKD healthcare professionals and the daily physical activity of CKD patients. It is a coaching training protocol that does not require extra equipment and could be used in the future as a non-invasive low-cost intervention for patients with CKD for improving their performance status and quality of life.

This scoping review was undertaken as a precursor to future systematic reviews that confirm the results shown here. In this regard, we performed a preliminary mapping of published literature that could be taken as a base for clinical practice. In addition, it is necessary to conduct future randomized controlled trials using different breathing training programs in the various CKD stages.
