# Supplementary material for: Role of breathing training programs on quality of life in chronic kidney disease patients
Source: AIMS Public Health. 2023 May 11;10(2):409–21. doi: 10.3934/publichealth.2023029 (PMC10251044; doi:10.3934/publichealth.2023029)
Supplement: Supplementary file 1 [file publichealth-10-02-029-s001.pdf]

Public Health

AIMS Public Health, 10(2): 409–421.
DOI: 10.3934/publichealth.2023029
Received: 18 November 2022
Revised: 07 May 2023
Accepted: 08 May 2023
Published: 11 May 2023

http://www.aimspress.com/journal/aimsph

Review

Role of breathing training programs on quality of life in chronic kidney disease patients

Ana I. Rubio-López[1], Alejandro Heredia-Ciuró[1], Jorge L. Marin-DelaRosa[2], Javier Martín-Núñez[1], María Granados-Santiago[1], María C. De Gracia-Guindo MD[2], and Marie C. Valenza PhD[1,*]

[1] Department of Physiotherapy, Faculty of Health Sciences, University of Granada, Granada, Spain
[2] Department of Nephrology, Virgen de las Nieves University Hospital, Granada, Spain

* **Correspondence:** Email: cvalenza@ugr.es; Tel: +34958248035.

Appendix A

(“Renal Insufficiency, Chronic” OR “Chronic Renal Insufficiencies” OR “Renal Insufficiencies, Chronic” OR “Chronic Renal Insufficiency” OR “Kidney Insufficiency, Chronic” OR “Kidney Insufficiencies, Chronic” OR “Chronic Kidney Insufficiency” OR “Chronic Kidney Insufficiencies” OR “Chronic Kidney Diseases” OR “Chronic Kidney Disease” OR “Disease, Chronic Kidney” OR “Diseases, Chronic Kidney” OR “Kidney Disease, Chronic” OR “Kidney Diseases, Chronic” OR “Chronic Renal Diseases” OR “Chronic Renal Disease” OR “Disease, Chronic Renal” OR “Diseases, Chronic Renal” OR “Renal Disease, Chronic” OR “Renal Diseases, Chronic”) AND (“Breathing exercise” OR “Respiratory exercises” OR “Respiratory therapy” OR Respiration OR Inhalation OR exhalation OR “Exp inspiratory capacity” OR “Inspiratory capacity” OR “Exp respiratory muscles” OR “Respiratory muscles” OR “Respirat* Therap*” OR “Respirat* Train*” OR “Respirat* Retrainin*” OR “Respirat* Exercise*” OR “Respirat* Resist*” OR “Respirat* Conditioning” OR “Respirat* Strength*” OR "Respirat* Weakness” OR “Respirat* Endurance muscle*” OR “Inspirat* Therap*” OR “Inspirat* Train*” OR “Inspirat* Retrainin*” OR “Inspirat* Exercise*” OR “Inspirat* Resist*” OR “Inspirat* Conditioning” OR “Inspirat* Strength*” OR “Inspirat* Weakness” OR “Inspirat* Endurance muscle*” OR “Expirat* Therap*” OR “Expirat* Train*” OR “Expirat* Retrainin*” OR “Expirat* Exercise*” OR “Expirat* Resist*” OR “Expirat* Conditioning” OR “Expirat* Strength*” OR “Expirat* Weakness” OR “Expirat* Endurance muscle*” OR “Ventilat* Therap*” OR “Ventilat* Train*” OR “Ventilat* Retrainin*” OR “Ventilat* Exercise*” OR “Ventilat* Resist*” OR “Ventilat* Conditioning” OR “Ventilat* Strength*” OR “Ventilat* Weakness” OR “Ventilat* Endurance muscle*” OR “Pulmonary Therap*” OR “Pulmonary Train*” OR “Pulmonary Retrainin*” OR “Pulmonary Exercise*” OR “Pulmonary Resist*” OR “Pulmonary Conditioning” OR “Pulmonary Strength*” OR

"Pulmonary Weakness" OR "Pulmonary Endurance" OR "Pulmonary muscle*" OR "Breathing exercise*" OR "Inhalation exercise*" OR "Exhalation exercise*" OR "Breathing therap*" OR "Inhalation therap*" OR "Exhalation therap*" OR "Breathing train*" OR "Inhalation train*" OR "Exhalation train*" OR "Breathing retrain*" OR "Inhalation retrain*" OR "Exhalation retrain*" OR "Breathing retrain*" OR "Inhalation retrain*" OR "Exhalation retrain*")

AIMS Press

© 2023the Author(s), licensee AIMS Press. This is an open access article distributed under the terms of the Creative Commons Attribution License (http://creativecommons.org/licenses/by/4.0)
